# Modeling the cost effectiveness of injury interventions in lower and middle income countries: opportunities and challenges

**DOI:** 10.1186/1478-7547-4-2

**Published:** 2006-01-19

**Authors:** David M Bishai, Adnan A Hyder

**Affiliations:** 1 Johns Hopkins University, Bloomberg School of Public, Department of Population and Family Health Sciences, 615 N. Wolfe Street, Baltimore, MD 21205, USA; 2 Johns Hopkins University, Bloomberg School of Public, Department of International Health, 615 N. Wolfe Street, Baltimore, MD 21205, USA

## Abstract

**Background:**

This paper estimates the cost-effectiveness of five interventions that could counter injuries in lower and middle income countries(LMICs): better traffic enforcement, erecting speed bumps, promoting helmets for bicycles, promoting helmets for motorcycles, and storing kerosene in child proof containers.

**Methods:**

We adopt an ingredients based approach to form models of what each intervention would cost in 6 world regions over a 10 year period discounted at both 3% and 6% from both the governmental and societal perspectives. Costs are expressed in local currency converted into US $2001. Each of these interventions has been assessed for effectiveness in a LMIC in limited region, these effectiveness estimates have been used to form models of disability adjusted life years (DALYs) averted for various regions, taking account of regional differences in the baseline burden of injury.

**Results:**

The interventions modeled in this paper have cost effectiveness ratios ranging from US $5 to $ 556 per DALY averted depending on region. Depending on local acceptability thresholds many of them could be judged cost-effective relative to interventions that are already adopted. Enhanced enforcement of traffic regulations is the most cost-effective interventions with an average cost per DALY of $64

**Conclusion:**

Injury counter measures appear to be cost-effective based on models. More evaluations of real interventions will help to strengthen the evidence basis.

## Background

Injuries are a growing global public health problem. Injuries killed over 5 million people in 2000 with many more being disabled, resulting in a heavy disease burden for people in all age categories [1]. For example, road traffic injuries drain developing economies of 1–2% of gross domestic product (about $100 billion) each year, or twice the total development aid received worldwide by developing countries [2].

Although high-income countries have had success in implementing and identifying effective injury interventions, few of these interventions have been tested in poorer countries. Interventions implemented in high-income countries are often thought to be beyond the capacity of low and middle-income countries. Some injury control policies require functioning legal institutions, enforcement personnel, and some require costly capital investments during road construction. The inequity in the feasibility of injury countermeasures around the world has caused concern, and lends further emphasis to the need for research on injuries in low and middle-income. [3,4]

In general, public efforts in injury control are poorly funded in LIMC [1,5]. The low expenditure compares unfavorably with other conditions and with that of more developed nations where government efforts for safety are well funded. Even adjusting for the 20–30 fold difference in GDP per capita between the developed nations and these poor countries, the investment disparities reflects that a low priority is given to safety in developing countries. Given the current low level of investment, initial investments in injury prevention and control if chosen with care, could turn out to be extremely beneficial to public health and welfare.

If cost-effectiveness analyses of injury interventions were able to document high returns they could help to encourage widespread efforts for implementation. Few if any injury counter measures in LMIC have been subjected to rigorous cost-effectiveness analysis[6]. One exception is a recent model of the costs and benefits of seat-belt enforcement in South Africa [7]. Furthermore, the scarcity of data on injury epidemiology and intervention efficacy in LMIC, makes it difficult to derive estimates of cost-effectiveness without building a model of the intervention and applying several assumptions. However, in view of the need for such data to inform decision making, and the opportunity to start assessing the potential benefit of investments in injury prevention, this paper will proceed to apply cost effectiveness analysis in this field. The main purpose of this paper is to stimulate an informed dialogue about the need for informing decisions in the health and allied sectors that improve health and human welfare by reducing the burden of injuries in LMIC.

## Methods

In this paper, we chose to model the costs and effectiveness of five interventions to prevent injury for which there was data on effectiveness in a LMIC context. These interventions, listed in Table 1, are: 1) improved enforcement of traffic codes; 2) building speed bumps at high risk intersections; 3) requiring and enforcing use of bicycle helmets in China; 4) requiring and enforcing use of motorcycle helmets in China; and 5) distributing childproof containers for paraffin/kerosene in South Africa. For two interventions, enforcement and speed bumps, we chose to model how the cost-effectiveness might differ in various world regions. For the other interventions, there was insufficient data on the epidemiology of injury to support extrapolation outside the country where the intervention was evaluated.

**Table 1:** Interventions Modeled in this Paper

**Intervention**	**Injury addressed**	**Type of intervention**	**Locus of intervention**	**Age group affected**
Improved enforcement	Road traffic injuries	Active	Traffic police	All
Speed bumps	Road traffic injuries	Passive	Transport, Civil Works	All
Bicycle helmets	Road traffic injuries	Active	Individuals	All; especially older children
Motorcycle helmets	Road traffic injuries	Active	Individuals	All, especially young adults
Childproof containers	Poisoning	Passive	Pharmaceutical companies	Children

A standard approach for intervention cost effectiveness analysis has been used in this paper following guidelines set up by a panel of economists working on the Disease Control Priorities Project [8]. All estimates of cost are presented in local currency converted to $ US (2001). The societal perspective is adopted for each intervention, and for the enforcement intervention, the government perspective is adopted.

To improve comparability, the time horizon for each intervention is taken to be one year of sustaining the intervention. Thus costs are annualized so that costs for a typical year of operating the intervention have been estimated. As with any intervention, there may be later reductions in annual operating costs as those who implement the intervention (staff) learn ways to do their tasks more efficiently.

Each year of program operation prevents an estimated number of deaths and injuries. In each case we present estimates of the raw numbers of deaths and the undiscounted numbers of life years this represents. However, from an economic perspective the life years and disability adjusted life years these persons enjoy in the far future count less. For comparability with other economic estimates we discount estimates of DALYs using both a 3% and a 6% discount rate. The 3% discount rate is based on costs of funds in higher income countries. However, there is a consensus that a higher discount rate may be appropriate in LMIC where there is a societal preference for more immediate consumption [9] To accommodate the possible appropriateness of higher discount rates a 6% discount rate is always presented.

## Results and discussion

The results of the cost effectiveness analysis are described below for each intervention, with descriptions of the types and considerations of costs, effectiveness and assumptions.

### Intervention 1: Improved enforcement of traffic codes

This intervention refers to enhanced enforcement of strengthened traffic regulations such as increased penalties for speeding and other proven effective road safety interventions, combined with media coverage.

#### Resource costs

This intervention requires three components: legislative change to achieve the stiffer penalties, media coverage of the new regime, and better enforcement.

##### Costs for legislative change

Legislative change imposes political costs in the sense that health advocates need to capture the attention of legislators and direct it to a particular issue. Although there are financial components to the process of legislative change, we assume the health sector is regularly monitoring and advocating its positions, and that focusing legislation on safer road transport does not create an incremental burden on this endeavor.

##### Costs for media coverage

Although it is conceivable that simply passing laws and enforcing them would be self-publicizing in some settings, the available programmatic literature evaluating an effort in Brazil where media promotion was an integral component of the strategy suggests that additional expenditure is likely to be required for media coverage [12]. Thus, we produce two models of cost-effectiveness, the baseline model with expenditure for media coverage, and an alternative model where the same effects are achieved without any direct spending on media coverage.

To estimate costs of media coverage we draw on the health communications literature. Estimates from the Philippines suggest that using television it costs $0.06 to reach one person, one time, and costs $0.10 to achieve recall [10]. Television media is appropriate because automobile drivers should be affluent enough to access television. Communications specialists estimate higher costs to achieve health behavior change, but their interventions seldom have an enforcement component to deliver immediate consequences to those who do not modify their behavior. There simply is no global database of media coverage costs by world region. We will therefore use wide confidence intervals from $0.01 per person reached, to $0.50 per person with recall in order to encompass the range that could prevail throughout the developing world.

##### Costs of better enforcement

Surprisingly, there are no published reports of the net costs of traffic enforcement from the government perspective. In fact, a recent review of road traffic interventions highlights this gap with special reference to the need for estimating such costs in LMIC [6].

From the societal perspective of traffic enforcement, both police salaries and motorists' citations count as opportunity costs – societal resources that could be used for other purposes. However, the perspective of the government is of fundamental importance to legislators deciding to enact traffic safety legislation. And from the government's perspective motorists' citations yield revenue, which is typically more than sufficient to defray the police costs of enforcement.

The State of Michigan (United States) is one of the few police units that publishes statistics on the economics of its traffic enforcement operations. In Michigan in 2000 there were 8.5 million vehicles in the state generating 93 billion vehicle miles traveled. In this same year Michigan employed 1,207 state police at 63 posts who generated $100 million in traffic citation revenue. The average citation in Michigan was $40 and about 1 of every 3 vehicles was cited during the year.

With a salary and fringe benefits totaling $50,000 per police officer, the salary costs of enforcement would be $60 million. Add an additional $30 million for vehicles and fuel, and another $10 million for collection costs, and the entire enforcement operation breaks even for a cost of $0 from the government's perspective. Yet from the societal perspective, the $100 million in citations are lost resources that are financed by citations.

We model costs of adequate enforcement in each world region by assuming the following:

• One officer per 5,000 vehicles, so that by ticketing 5–10 per day the officer would cite about 1 of every 3 vehicles per year.

• The officer would draw the salary of a level 3 employee [11]. Freelance citations are subsumed in salary up to a maximum of level 3 salary. In other words in a system that pays police less than a level 3 salary, the police supplement their income up to a level 3 salary by issuing freelance citations which they keep as income.

• One police vehicle shared by every 2 officers

• Motorists' costs of paying citations are not included in societal costs because that would be double counting the resources used to provide traffic enforcement.

• Prior to the intervention, police strength is 50% of the level of adequacy defined above. Prior to the intervention officers are writing citations at full capacity so that the only way to increase enforcement is to hire additional traffic police to enable citation of 1 of every 3 vehicles.

We use data from World Road Indicators to estimate the number of vehicles per million persons in each world region to complete our cost estimates in Table 2[12].

**Table 2:** Cost of treating community of 1 million with increased traffic penalties, enforcement, and a media campaign

*Region*	Vehicles per million persons**	*Target number of traffic police per million* * persons @ 1 officer per 5000 vehicles†*.	Police costs only*	*Media costs only*	*Enforcement costs plus media costs*
EAP	16,000	3	$10,217	$1,600	$11,817
ECA	204,000	41	$175,571	$20,400	$195,971
LAC	158,000	32	$209,713	$15,800	$225,513
MENA*	57,000	11	$109,215	$5,700	$114,915
SA	8,000	2	$7,305	$800	$8,105
SSA*	24,000	5	$22,118	$2,400	$24,518
**Un weighted Average**			$89,023	$7,783	$96,807

*Data are from 1990

**Source: World Development Indicators 2003, World Bank, 2003.

† Figures pertain to the minimum number of police officers required to issue citations to 1/3 of the 5000 vehicles in their beat each year. Assume baseline staffing is 50% of numbers given in column 3 and assume every 2 officers require 1 police vehicle.

Notes: EAP: East Asia & Pacific, ECA: Europe & Central Asia, LAC: Latin America & Caribbean, MENA: Middle East & North Africa, SA: South Asia, SSA: Sub-Saharan Africa.

#### Outcome

According to a Brazilian study intervening with these three ingredients achieved a 25% reduction in traffic fatalities between 1997 and 1998 [13]. We model the number of lives saved by assuming that the Brazilian experience of 25% reductions from baseline traffic deaths would be observed in each world region. There simply are not region specific evaluations of this intervention with which to challenge this assumption. Readers will have to judge whether in fact, the Brazilian results can actually be extrapolated to a particular community where the epidemiology and culture could differ markedly (Table 3).

**Table 3:** Cost per death averted of treating community of 1 million with better enforcement

*Region*	*Government perspective:* * No enforcement costs; media costs only*	*Societal perspective:* * Enforcement only*	*Societal perspective:* * Enforcement costs plus media costs*
EAP	$35	$221	$256
ECA	$468	$4,027	$4,495
LAC	$390	$5,171	$5,560
MENA	$86	$1,657	$1,744
SA	$17	$157	$174
SSA	$34	$313	$347
**Unweighted Average**	$172	$1,924	$1,803

Based on data from World Road Statistics, traffic fatalities in LMICs occur in a ratio of 8 non-fatal injuries per fatality [14]. Of these non-fatal injuries, on the average, 10% will incur permanent disability, with a severity that translates to a disability weight of 0.3. [15]. Traffic deaths are assumed to occur at a mean age of 20 years.

Thus each fatality accounts for 1 year of life lost due to death plus disability adjusted life years (YLD) for the 8 injured people (Table 4). We multiply the 8 injured persons by the 10% for those permanently disabled of all injured and then adjust it for a disability severity of 30% for permanently disabled persons for every year lived in the life expectancy (or 8 × 0.1 × 0.3). The average Life Expectancy at 20 years of age is roughly 50 years in every region except sub Saharan Africa, where we use the estimate of 37 years (Table 5). Finally all DALYs in our estimates are discounted at both 3% and at 6%. Finally we present the costs per DALY first discounted at 3% (Table 5) then discounted at 6% (Table 6).

**Table 4:** Cost per undiscounted life year saved of treating community of 1 million with better enforcement

*Region*	*Government perspective:* * No enforcement costs; media costs only*	*Societal perspective:* * Enforcement only*	*Societal perspective:* * Enforcement costs plus media costs*
EAP	$0.69	$4	$5.12
ECA	$9.36	$81	$90
LAC	$7.79	$103	$111
MENA	$1.73	$33	$35
SA	$0.34	$3	$3
SSA	$0.92	$8	$9
**Unweighted Average**	$3.47	$39	$42

**Table 5:** Cost per DALY DISCOUNTED AT 3% saved from treating community of 1 million with better enforcement

*Region*	*Government perspective:* * No enforcement costs; media costs only*	*Societal perspective:* * Enforcement only*	*Societal perspective:* * Enforcement costs plus media costs*
EAP	$1.05	$7	$8
ECA	$14.24	$123	$137
LAC	$11.85	$157	$169
MENA	$2.63	$50	$53
SA	$0.52	$5	$5
SSA	$1.20	$11	$12
**Unweighted Average**	$5.25	$59	$64

**Table 6:** Cost per DALY DISCOUNTED AT 6% saved from treating community of 1 million with better enforcement

*Region*	*Government perspective:* * No enforcement costs; media costs only*	*Societal perspective:* * Enforcement only*	*Societal perspective:* * Enforcement costs plus media costs*
EAP	$1.67	$11	$12
ECA	$22.59	$194	$217
LAC	$18.80	$250	$268
MENA	$4.17	$80	$84
SA	$0.83	$8	$8
SSA	$1.75	$16	$18
**Unweighted Average**	$8.30	$93	$101

With deaths averted and DALYs averted as the outcomes, it would be desirable to be able to calculate the savings of medical care prevented from fewer non-fatal crashes directly attributable to this intervention. However, there is no "typical" medical treatment for traffic related crash morbidity. Furthermore there is too little data on the incidence and severity of non-fatal crashes. This presents an analytical dilemma, because it would be misleading to exclude savings from preventing non-fatal crashes, and it would be misleading to fabricate an estimate of the magnitude of the savings by region.

We present a partial resolution by building on an in-depth case study of the cost of road crashes in Bangladesh [16] where it was estimated that for every fatal crash in 2002, there were 36 non fatal crashes: 8 of which were serious and 28 with slight injuries.

The costs for these injuries could be broken down as shown in Table 7.

**Table 7:** Medical and Non Medical Costs of Injury

	Property	Administration	Lost output	Medical cost	Human cost*	**Total cost**
Serious	$975	$17	$316	$357	$351	**$2,016**
Slight	$690	$17	$32	$36	$155	**$929**

*Source: Ross Silcock and TRL 2003. Data are for Bangladesh, year 2002. Taka converted to U.S. dollars at 60 Taka = $1 dollar.

** Human costs pertain to pain and suffering

Thus, if one prevented traffic fatality was associated with preventing 8 serious crashes worth (8 × $2,016 =) $16,128 and preventing 28 slight injuries worth (28 × $929 =) $26,012 then there would be an additional $42,140 in total cost savings associated with preventing the non-fatal events that one can assume occur in the system per every fatal event.

If the enforcement costs in Bangladesh are close to the $8,105 listed in Table 2, then the intervention would save more money than it cost if it only prevented one death. If an enforcement intervention in Bangladesh is as effective as the one documented in Brazil, it could lower fatalities by 25% [13]. With 83 traffic fatalities per million population, the intervention could prevent 21 deaths and lead to a net savings of ($8,105 - 21 × $42,140 =) -$876,835 saved for every million population receiving this intervention.

Because of inadequate data on the burden of non-fatal crashes, we have only included an analysis of cost offsets for the case of Bangladesh. It is entirely possible that substantial savings also occur in other countries and regions. Collecting the data to permit such an analysis in other regions should be a priority.

### Intervention 2: speed bumps

#### Resource Costs

We estimate costs (Table 8) by assuming the following:

**Table 8:** Number of speed bumps targeted and cost of constructing speed bumps

*Region*	*Number of fatalities* at junctions*	*Number of junctions accounting* for 10% of fatalities**	*Number of junctions accounting* * for 25% of fatalities*	*Annualized Cost to Treat 1 Junction* * with Speed Bumps*
EAP	92	3	9	$223
ECA	87	3	8	$230
LAC	81	3	8	$105
MENA	132	5	13	$231
SA	93	3	9	$99
SSA	141	6	14	$100
**Unweighted Average**				$165

*In an urban population of 1 million, assuming that 50% of traffic deaths occur at junctions.

**Assuming degree of hazard at junctions is distributed exponentially. Figures are rounded to nearest whole number.

• In an urban population, 50% of traffic fatalities are due to crashes at junctions.

• The degree of hazard of a city's junctions is distributed as a negative exponential, with a few very hazardous ("black spot") junctions accounting for multiple deaths per year, and a long list of other junctions that are associated with a death less than or up to once a year.

• The top decile of hazardous junctions would be amenable to treatment with speed bumps

• The cost of constructing a speed bump in Africa is $1,000 [17]

• Relative costs of constructing speed bumps by region are the same as relative costs of constructing buildings; thus we extrapolate the African costs to the other world regions by the relative building cost

• A speed bump is assumed to last 10 years before it must be reconstructed. We assume linear depreciation. Thus 1 year of speed bump services in Africa cost $100.

##### Outcome

Table 9 presents our estimates of cost per death averted based on the assumptions described above and costs in Table 8. Three variants of treating junctions with speed bumps have been modeled.

**Table 9:** Cost per death averted of treating high risk junctions with speed bumps

*Region*	*Treating a single black spot junction* * with 4 annual fatalities*	*Treating the most dangerous junctions* * causing 10% of junction deaths*	*Treating the most dangerous junctions* * causing 25% of junction deaths*
EAP	$101	$143	$156
ECA	$105	$147	$161
LAC	$48	$67	$73
MENA	$105	$148	$161
SA	$45	$63	$69
SSA	$45	$64	$70
**Unweighted Average**	$75	$105	$115

To compute life years saved in Table 10, we assume that the deaths averted due to traffic crashes occur at age 20. Regional LE-20 estimates are used as before, and the three types of treating junctions are modeled.

**Table 10:** Cost per undiscounted life year saved of treating high risk junctions with speed bumps

*Region*	*Treating a single black spot junction* * with 4 annual fatalities*	*Treating the most dangerous junctions* * causing 10% of junction deaths*	*Treating the most dangerous junctions* * causing 25% of junction deaths*
EAP	$2.03	$2.85	$3.12
ECA	$2.09	$2.94	$3.22
LAC	$0.95	$1.34	$1.46
MENA	$2.10	$2.95	$3.22
SA	$0.90	$1.27	$1.39
SSA	$1.23	$1.73	$1.89
**Unweighted Average**	$1.55	$2.18	$2.38

Finally, the cost per discounted DALY using both 3% (Table 11) and 6% (Table 12) are presented for the three models.

**Table 11:** Cost per DALY DISCOUNTED AT 3% of treating high risk junctions with speed bumps

*Region*	*Treating a single black spot junction* * with 4 annual fatalities*	*Treating the most dangerous junctions* * causing 10% of junction deaths*	*Treating the most dangerous junctions* * causing 25% of junction deaths*
EAP	$3.09	$4.34	$11.85
ECA	$3.18	$4.48	$12.23
LAC	$1.45	$2.04	$5.57
MENA	$3.19	$4.49	$12.26
SA	$1.37	$1.93	$5.27
SSA	$1.61	$2.26	$6.17
**Unweighted Average**	$2.31	$3.26	$8.89

**Table 12:** Cost per DALY DISCOUNTED AT 6% of treating high risk junctions with speed bumps

*Region*	*Treating a single black spot junction* * with 4 annual fatalities*	*Treating the most dangerous junctions* * causing 10% of junction deaths*	*Treating the most dangerous junctions* * causing 25% of junction deaths*
EAP	$4.89	$6.89	$18.80
ECA	$5.05	$7.11	$19.40
LAC	$2.30	$3.23	$8.83
MENA	$5.06	$7.12	$19.45
SA	$2.18	$3.06	$8.36
SSA	$2.35	$3.30	$9.01
**Unweighted Average**	$3.64	$5.12	$13.98

### Intervention 3: bicycle helmet legislation and enforcement

Baseline epidemiological data on the burden of bicycle injuries is a prerequisite to estimating the deaths averted in a region. Because bicycle ridership is quite variable across regions, it is impossible to extrapolate estimates of the epidemiological burden. We are fortunate to have an in-depth report for China where bicycle related deaths kill 22 per 1,000,000 per year [18]. Until more epidemiological data is collected on bicycle crashes, we only feel confident making estimates of potential bicycle helmet cost-effectiveness for China.

#### Resource costs

We assume the following for modeling bicycle helmet costs:

• No financial cost to society of passing new legislation.

• New enforcement costs are small. Police need to cite only 1% of bicyclists per year to achieve and maintain high compliance. Unlike other traffic violators, helmet-less riders know that they are very easy to detect at all times that they are on the road.

• On foot, one police officer can cite 2,500 helmet violators in a year.

• Costs for the police officer are a salary of a level 3 worker.

• Citations exactly pay for the officer's salary, but count as an additional societal cost.

• One helmet in China costs $10.00 in local currency converted to $US circa 2001 [19]. Although for leisure bicycling, one helmet may be used by more than one bicyclist, we assume a case of business commuting in China implying a one helmet to one bicyclist ratio.

• Each helmet lasts 10 years with linear depreciation, making one helmet-year cost $1

• We ignore savings from prevented medical spending.

In a population of 1 million we assume that there are 250,000 regular bicyclists, which will require the equivalent of 1 full time police officer in order to cite 1% of them for helmet violations. At Chinese salary levels this would cost the equivalent of $15,000.

The helmets for this population would cost $250,000 at $1 per year of helmet use. Thus the total cost of the intervention would be $265,000.

#### Outcome

The population based death rate from bicycle injuries is 22 per million in the province of Wuhan. We assume that in a population of 1 million there would also be at least 220 coincident head injuries, although this may well be an underestimate. A case control study showed that bicycle helmet use was associated with an 85% reduction in relative risk of head injury [20]. We assume that the 85% reduction would apply to bicycle deaths as well, although this may be a slight over-estimate. Thus if a population makes a transition from zero helmets to 100% compliance it would avert 0.85 × 22 = 19 deaths and 190 survivable head injuries.

We assume a mean age of injury of 20 years so that each victim loses a flow of 50 years of life discounted at 3% and 6% if they die. We also assume a disability weight of 0.4 lost YLD per year spent with brain injury based on the long term WHO disability weights [21].

Thus achieving full compliance an intervention to increase helmet use that costs $2,515,000 can prevent (19 × PV(50)) + (190 × PV(0.4 × 50)) DALYS where PV represents the "present value" function discounting at 3% or 6%. The DALYs gained amount to 2,478 and 1,562 at 3% and 6% discount rates respectively. Thus the cost effectiveness of going from 0 to 100% helmet use in China would be $107 per DALY (= $265,000/2478) or $170 per DALY (= $265,000/1562) at 3% and 6% discount rates respectively.

### Intervention 4: motorcycle helmet legislation and enforcement

As with bicycles, we are fortunate to have epidemiological data for China where motorcycle related deaths kill 16 per 1,000,000 per year [22]. Thus, we only feel confident making estimates of potential helmet cost-effectiveness for China. The assumptions about the cost of a motorcycle intervention are generally the same as those for bicycle helmets (see above).

#### Resource costs

We assume the following to model costs of motorcycle helmet legislation:

• No financial cost to society of passing the new legislation (in China such legislation already exists).

• Police need to cite only 1% of motorcyclists per year to achieve and maintain high compliance.

• On foot, one police officer can cite 2,500 helmet violators in a year.

• Costs for the police officer are a salary of a level 3 worker [8].

• Citations exactly pay for the officer's salary, but count as an additional societal cost.

• One motorcycle helmet in China costs $20.00 and lasts 10 years with linear depreciation leading to $2.00 per year of use.

• We ignore medical cost savings from less severely injured patients.

In a population of 1 million we assume that there are 125,000 regular motorcyclists, which will require the equivalent of 1/2 full time police officer in order to cite 1% of them for helmet violations. At Chinese salary levels this would cost the equivalent of $7,500. The helmets for this population would cost $250,000 at $2 per year of helmet use. Thus the total cost of the intervention would be $257,500.

#### Outcome

The population based death rate from motorcycle injuries is 16 per million [22]. We assume that in a population of 1 million there would also be 160 coincident head injuries, although this is likely to be an underestimate. Data from Thailand indicates that following legislation and enforcement, head injuries decreased by 41% and deaths by 21% [23].

Thus, applying the Thai experience, if a population enacts motorcycle helmet legislation and enforcement it could prevent, say 21% × 16 deaths and 41% × 160 head injuries. Assuming a mean age of injury of 20 years, and a disability weight of 0.4 for head injury, we can estimate discounted DALYs achieved through motorcycle helmet legislation as 784 and 495 at respective discount rates of 3% and 6%. This intervention thus costs $467 per DALY (= $275,000/784) or $769 per DALY (= $275,000/495) based on 3% or 6% discount rates respectively.

### Intervention 5: Childproof containers for paraffin (kerosene)

This intervention is relevant to regions, such as sub-Saharan Africa, where paraffin (kerosene) is used as a cooking fuel and is frequently stored in bottles similar to those used to store beverages [24,25]. A series of studies from South Africa has significantly enhanced our understanding of the cost-effectiveness of intervening for this problem by distributing Child Resistant Containers (CRCs).

#### Resource costs

Based on reports from South Africa we assume the following to model costs of CRCs:

• In a population of 1 million, CRCs would need to be distributed to 200,000 households

• Each CRC cost 0.85 Rand ($0.33) including costs of distribution

• Total direct costs would be 200,000 × $0.33 = $66,000

The intervention to prevent poisoning would prevent hospitalizations and generate savings to the medical sector. In a population of 1 million total population who used paraffin regularly, the South African experience was 1,040 annual poisonings at baseline. After CRCs were distributed the incidence would drop to 540 annual poisonings, indicating that 500 annual poisonings would be prevented [25]. In South African hospitals the average cost for a poisoned child was 256.13 Rand ($100) per child. So indirect cost savings would be 500 × $100 = $50,000 which would partially offset the $66,000 direct costs leading to a total cost of $16,000 to intervene.

#### Outcome

The mean age of children who suffered poisoning in South Africa was 12–24 months. There were no deaths among children in the South African study but the most common report in the literature is a 2% case fatality rate [25] suggesting that having prevented 500 poisonings one would prevent 10 deaths occurring around age 2. Based on the life tables for sub-Saharan Africa, life expectancy at age 2 is 49 years [8]. Thus, 490 life years could be saved by the $16,000 intervention.

As a result, one estimate of the cost-effectiveness of CRCs as a method to stop paraffin poisoning in South Africa would be $16,000/10 = $1600 per death averted. Most survivors of paraffin poisoning do not suffer permanent disability, and lacking any objective means to assign disability weights to those who are disabled, we neglect YLDs in calculating DALYs. The investment of $16,000 thus produces 10 children surviving for 49 more years. Undiscounted this is 10 × 49 = 490 years. With discounting the impact is 263 DALYs averted and 166 DALYs averted discounted at 3% and 6% respectively. The cost-effectiveness is $61 per DALY (= 16,000/263) at 3% or $96 per DALY (= 16,000/166) at 6% making it one of the most highly cost-effective interventions we have considered.

## Conclusion

Arguments using the value of investments for specific interventions are used every day to assess the rationale for investing in the prevention and control of diseases. The field of injury prevention has lacked such estimates for low and middle income countries. The reasons for lack of such estimates include the relative under-investment in injury research, lack of recognition of injuries as a public health problem, and lack of appropriate capacity in the developing world [26,27]. This creates a classic dilemma – do we wait for intervention trials in low and middle income countries prior to embarking on such estimates, or do we go ahead and make best use of available data using transparent and explicit assumptions to model costs and effectiveness of interventions? This paper will put forward cost effectiveness estimates based on models of five specific interventions in order to make the most of the few situations where there have actually been effectiveness studies in LMIC.

All of the interventions modeled in this paper are relatively cost effective ranging from US$ 5 to 556 per DALY (discounted at 3%). For the two interventions modeled in different regions of the world, the geographical variation was highest between the most developed region (ECA) and the developing regions (SA, SSA) rather than in between the lower income regions. It is unclear what proportion of this variation is due to differences in the assumptions used by region, versus real differences based on the burden of disease. It is also clear that the rate of discounting also affects (often doubles) the cost-effectiveness estimate and so is an important factor in both estimation and presentation of results for comparison across interventions.

Enhanced speeding control is a strongly supported as a recommendation of the World Report on Road Traffic Injury Prevention [27] and is highly cost effective at $93 per DALY discounted at 6%. At US$13.98 per DALY, treating 25% of the most dangerous junctions with speed bumps turns out to be highly cost effective, but requires the identification of such intersections. Bicycle helmet legislation has not been universally implemented in the developed world as yet, and yet a cost effectiveness of $170/DALY (discounted at 3%) makes them attractive for further consideration. Interestingly, motorcycle helmet legislation in East Asia was found to have a higher cost and lower benefit at $556/DALY (discounted at 3%) with the important proviso that this model assumed that motorcycle helmets cost twice what bicycle helmets cost and was based on evidence that motorcycle helmets were less effective than bicycle helmets in preventing head injuries and deaths. Finally at $96/DALY (discounted 3%) the child resistant containers are a highly cost effective intervention for serious consideration by a large part of the developing world where paraffin (kerosene) is used; including sub-Saharan Africa, South Asia, East Asia and parts of the Middle East.

The challenges of assessing injury interventions have not prevented us from moving forward. The assumptions used in this paper have been transparently described and influence the results. Our work was limited by the availability of injury and cost data. Researchers are invited to discuss and explore alternate assumptions, additional data and models to generate more refined or additional estimates. Such exploration and interest would achieve the larger objective of furthering interest in mapping injury interventions.

## List of Abbreviations

LMIC: Low and middle income country

DALY: Disability adjusted life year

YLD: Year of life lived with disability

PV: Present value

CRC: Child resistant container

EAP: East Asia and Pacific

ECA: Europe Central Asia

LAC: Latin America and Caribbean

MENA: Middle East and North Africa

SA: South Asia

SSA: Sub Saharan Africa

## Competing interests

The author(s) declare that they have no competing interests.

## Authors' contributions

AH and DB jointly conceived the study and identified the interventions for study. DB carried out the economic modelling. AH and DB jointly authored the paper and the interpretation of the findings.
